# The unexpected role of polyubiquitin chains in the formation of fibrillar aggregates

**DOI:** 10.1038/ncomms7116

**Published:** 2015-01-20

**Authors:** Daichi Morimoto, Erik Walinda, Harumi Fukada, Yu-Shin Sou, Shun Kageyama, Masaru Hoshino, Takashi Fujii, Hikaru Tsuchiya, Yasushi Saeki, Kyohei Arita, Mariko Ariyoshi, Hidehito Tochio, Kazuhiro Iwai, Keiichi Namba, Masaaki Komatsu, Keiji Tanaka, Masahiro Shirakawa

**Affiliations:** 1 Department of Molecular Engineering, Graduate School of Engineering, Kyoto University, Kyoto-Daigaku Katsura, Nishikyo-Ku, Kyoto 615-8510, Japan; 2 Graduate School of Life and Environmental Sciences, Osaka Prefecture University, Naka-ku, Sakai, Osaka 599-8531, Japan; 3 Protein Metabolism Project, Tokyo Metropolitan Institute of Medical Science, Setagaya-ku, Tokyo 156-8506, Japan; 4 Department of Biochemistry, School of Medicine, Niigata University, Chuo-ku, Niigata 951-8510, Japan; 5 Graduate School of Pharmaceutical Sciences, Kyoto University, 46-29 Yoshida-Shimoadachi, Sakyo-ku, Kyoto 606-8501, Japan; 6 Quantitative Biology Center, RIKEN, 1-3 OLABB, Osaka University 6-2-3, Furuedai, Suita, Osaka 565-0874, Japan; 7 Laboratory of Protein Metabolism, Tokyo Metropolitan Institute of Medical Science, Setagaya-ku, Tokyo 156-8506, Japan; 8 Graduate School of Medical Life Science, Yokohama City University, 1-7-29 Suehiro-cho, Tsurumi-ku, Yokohama, Kanagawa 230-0045, Japan; 9 Department of Biophysics, Graduate School of Science, Kyoto University, Oiwake-cho, Kitashirakawa, Sakyo-Ku, Kyoto 606-8502, Japan; 10 Graduate School of Medicine, Kyoto University, Yoshida-Konoe-cho, Sakyo-ku, Kyoto 606-8501, Japan; 11 Graduate School of Frontier Biosciences, Osaka University, 1-3 Yamadaoka, Suita, Osaka 565-0871, Japan

## Abstract

Ubiquitin is known to be one of the most soluble and stably folded intracellular proteins, but it is often found in inclusion bodies associated with various diseases including neurodegenerative disorders and cancer. To gain insight into this contradictory behaviour, we have examined the physicochemical properties of ubiquitin and its polymeric chains that lead to aggregate formation. We find that the folding stability of ubiquitin chains unexpectedly decreases with increasing chain length, resulting in the formation of amyloid-like fibrils. Furthermore, when expressed in cells, polyubiquitin chains covalently linked to EGFP also form aggregates depending on chain length. Notably, these aggregates are selectively degraded by autophagy. We propose a novel model in which the physical and chemical instability of polyubiquitin chains drives the formation of fibrils, which then serve as an initiation signal for autophagy.

## Abstract (web summary)

Ubiquitin is a stable and soluble protein, but it is commonly found in inclusion bodies in neurodegenerative disorders and cancer. Here, Morimoto *et al*. report that increasing ubiquitin chain length leads to the formation of amyloid-like fibrils, which are degraded by an autophagy mechanism.

## Graphical abstract

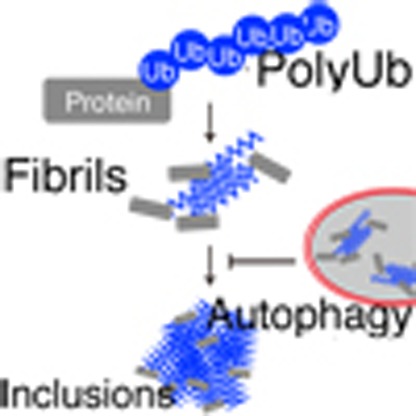

Ubiquitin is a small protein of 76 amino acids that folds into a compact globular structure. While researchers working in the field of protein chemistry widely use it as a model protein^1^,^2^, this protein has unique physicochemical and biological properties. For example, the tertiary structure of ubiquitin is known to be one of the most rigid among eukaryotic intracellular proteins, being much more tolerant to extreme pH changes and high temperature than most cellular proteins.

Ubiquitin exerts its biological functions when it is covalently conjugated to intracellular proteins in a highly specific manner. Like phosphorylation, ubiquitylation (the modification of proteins with ubiquitin) is prevalent in both normal and pathological cellular processes^3^,^4^ and is achieved by the successive action of ubiquitin-activating (E1), ubiquitin-conjugating (E2) and ubiquitin-ligating (E3) enzymes. Ubiquitin is first activated through the formation of an E1-ubiquitin thioester in an ATP-dependent manner and then transferred to an E2 enzyme via a thioester linkage. Finally, E3 enzymes participate in the formation of an isopeptide bond between a lysine residue on a substrate protein and the C-terminal tail of ubiquitin. This signal tag is recognized by downstream proteins containing a ubiquitin-binding domain (UBD) and can be removed from the target proteins by deubiquitinating (DUB) enzymes, which counterbalance the action of the E1–E2–E3 machinery in the cell^3^.

Ubiquitin can be attached to substrates either as a single moiety (monoubiquitylation) or as several independent ubiquitin molecules (multiple monoubiquitylation). In addition, multiple ubiquitin moieties can be covalently linked via their amino (N) terminus or any of the seven lysine (K6, K11, K27, K29, K33, K48 and K63) residues on a given ubiquitin and the carboxy (C) terminus of the next ubiquitin molecule, thereby forming polymeric ubiquitin chains. These chains have mixed topology if different linkages are formed at successive positions of the chain. The most extensively characterized forms of these polymers are ubiquitin chains linked through either K48 or K63. K48-linked polyubiquitylation predominantly targets proteins for proteasomal degradation, whereas K63-linked polyubiquitylation seems to regulate protein function, subcellular localization and protein–protein interactions^3^. The roles of other types of ubiquitin chain have not been studied in great detail, but data on their specific functions are just starting to emerge, suggesting that ubiquitylation can act as a code to store and transmit information by means of specific recognition by downstream ubiquitin-binding proteins of polyubiquitin chains and/or substrate proteins^3^,^5^.

Although exceptionally rigid and highly soluble *in vitro*, ubiquitin has been identified as a major component of protein inclusion bodies—intracellular aggregates that are associated with various intractable diseases including cancer and neurodegenerative disorders such as Alzheimer’s disease, Huntington’s disease and amyotrophic lateral sclerosis^6^,^7^,^8^. However, the formation mechanism and function of such ubiquitin-positive inclusion bodies have remained unclear ever since ubiquitin was first identified as a component of paired helical filaments in Alzheimer’s disease more than 25 years ago^7^. Recently, it has been proposed that ubiquitin-positive aggregates are selectively degraded by macroautophagy (hereafter referred to as autophagy), in which isolation membranes engulf cytoplasmic constituents and the resulting autophagosomes fuse with lysosomes, leading to degradation of their constituents^9^. However, the molecular mechanism by which the aggregates are specifically degraded by autophagy is largely unknown.

Herein, we propose a novel role of ubiquitylation termed ‘ubiquitin fibrils’. We show that the stability of ubiquitin is lost when ubiquitin chains are elongated, regardless of the type of ubiquitin chain. This results in the formation of polyubiquitin fibrils, which then act as a signal for clearance by autophagy.

## Results

### Longer ubiquitin chains have lower thermodynamic stability

**Figure 1:**
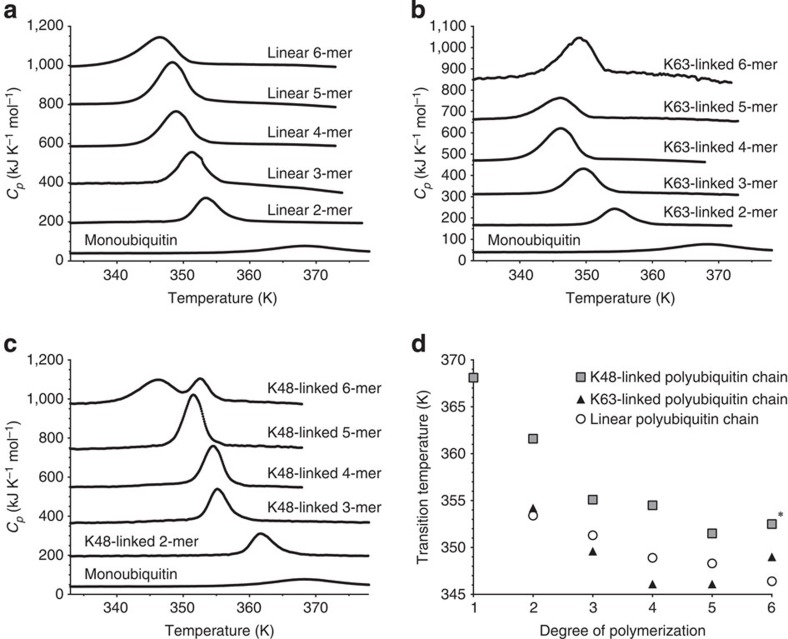
Comparative analysis of the thermal denaturation for polyubiquitin chains of different length. Differential scanning calorimetry traces of monoubiquitin and linear polyubiquitin chains (**a**), K63-linked polyubiquitin chains (**b**) and K48-linked polyubiquitin chains (**c**) with up to six ubiquitin units. (**d**) Transition temperatures are plotted against chain length for K48-linked, K63-linked and linear ubiquitin chains. (*In the thermal denaturation of K48-linked hexaubiquitin, the higher transition temperature is selected.).

We first found that the stability of ubiquitin chains significantly decreases with increasing chain length. As reported previously, monoubiquitin is highly heat stable^10^; here, differential scanning calorimetry (DSC) analysis showed that its transition temperature is 368 K. However, all polyubiquitin chains linked through Met 1 (linear), Lys 48 (K48) or Lys 63 (K63) have a transition temperature that is more than 15 K lower (Fig. 1a–c). Interestingly, longer chains show lower transition temperatures regardless of the linkage type (Fig. 1d and Supplementary Fig. 1).

**Figure 2:**
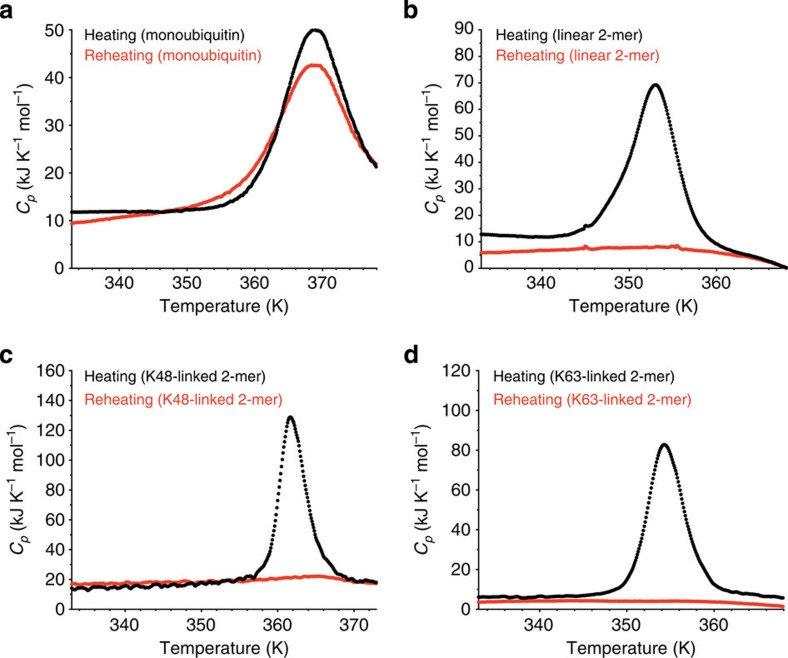
Irreversible thermal unfolding of diubiquitin chains. Differential scanning calorimetry traces of monoubiquitin (**a**), linear diubiquitin (**b**), K48-linked diubiquitin (**c**) and K63-linked diubiquitin (**d**) are shown. Black traces show initial heating and red lines represent reheating. Thermal unfolding of monoubiquitin is reversible, whereas thermal unfolding of diubiquitin is irreversible.

In addition, while the thermal transition of monoubiquitin is reversible, those of polyubiquitin chains are irreversible, as revealed by the DSC curves observed for reheating of the heat-denatured products of ubiquitin and its polymers. Ubiquitin is soluble at and above the transition temperature (Fig. 2a), whereas polyubiquitin chains exist as insoluble small aggregates above the transition temperatures that remain insoluble even when the temperature decreases below the transition temperatures (Fig. 2b–d). These observations suggest that conjugation of ubiquitin to another ubiquitin molecule impairs its ability to refold into its native structure, and that the addition of further ubiquitin molecules leads to the transition of ubiquitin from a native state to an aggregated state at even lower temperatures.

### Polyubiquitin chains form amyloid-like fibrils by heat

**Figure 3:**
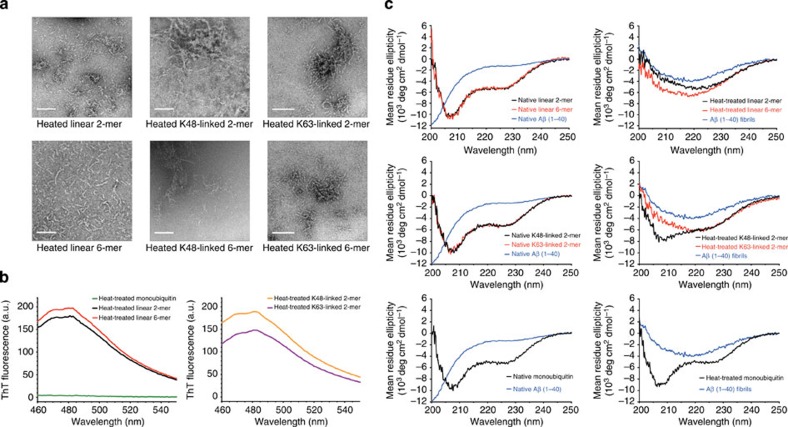
Heat-treated polyubiquitin chains form amyloid-like fibrils. (**a**) Electron microscopy (EM) images of heat-treated linear diubiquitin (left upper), linear hexaubiquitin (left lower), K48-linked diubiquitin (middle upper), K48-linked hexaubiquitin (middle lower), K63-linked diubiquitin (right upper) and K63-linked hexaubiquitin (right lower). Scale bar, 100 nm. (**b**) Thioflavin T fluorescence emission spectra of heat-treated monoubiquitin (left, green), linear diubiquitin (left, black), linear hexaubiquitin (left, red), K48-linked diubiquitin (right, orange) and K63-linked diubiquitin (right, purple). (**c**) Left, circular dichroism spectra of native linear diubiquitin (upper, black), hexaubiquitin (upper, red), K48-linked diubiquitin (middle, black) and K63-linked diubiquitin (middle, red), monoubiquitin (lower, black) and Aβ (1–40) (blue). Right, circular dichroism spectra of these heat-treated samples and Aβ (1–40) fibrils (blue).

Unexpectedly, we found that the aggregates formed by ubiquitin chains are amyloid-like fibril assemblies. The electron microscopy (EM) images of the aggregates formed by heating linear, K48-linked and K63-linked hexaubiquitin showed that they consist of fibrils of up to 100 nm in length and ~5 nm in diameter (Fig. 3a). Similar fibrils were also formed by heating linear, K48-linked and K63-linked diubiquitin but not by heating monoubiquitin (Fig. 3a and Supplementary Fig. 2a). The morphology of these fibrils was reminiscent of amyloid-like fibrils^11^; furthermore, the polyubiquitin fibrils were all stained with Thioflavin T (ThT), the binding and fluorescence of which are considered to be indicative of amyloid-like fibrils (Fig. 3b). Circular dichroism (CD) spectra revealed that the polyubiquitin aggregates consistently displayed β-rich secondary structure, highly similar to that observed in amyloid-β (Aβ) (1–40) fibrils (Fig. 3c), supporting the idea that polyubiquitin chains form amyloid-like fibrils.

Collectively, these observations indicate that heat denaturation causes the irreversible transition of polyubiquitin chains to amyloid-like fibrils and that longer chains form fibrils at lower temperatures. For other fibrillogenic proteins, a decrease in thermodynamic stability correlates closely with a tendency towards fibril formation^12^,^13^, which is consistent with the observed fibril formation of polyubiquitin chains.

### Shear forces can induce polyubiquitin fibril formation

**Figure 4:**
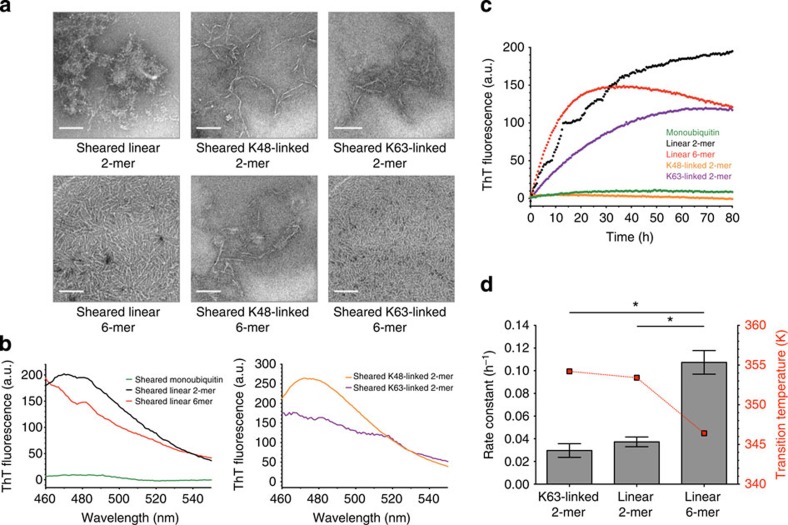
Mechanical stress induces the formation of ThT-positive fibrils of polyubiquitin chains. (**a**) EM images of sheared samples: linear diubiquitin (left upper), linear hexaubiquitin (left lower), K48-linked diubiquitin (middle upper), K48-linked hexaubiquitin (middle lower), K63-linked diubiquitin (right upper) and K63-linked hexaubiquitin (right lower) show that all of these types of polyubiquitin form fibrils with amyloid-like morphology when subjected to mechanical stress. The shear stress was applied as an agitation at a rotational speed of 25 or 33 s^−1^. Scale bar, 100 nm. (**b**) Thioflavin T fluorescence emission spectra of sheared monoubiquitin (left, green), linear diubiquitin (left, black), linear hexaubiquitin (left, red), K48-linked diubiquitin (right, orange) and K63-linked diubiquitin (right, purple). Shear stress was applied as an agitation at a rotational speed of 25 s^−1^ for 90 h. For monoubiquitin and K48-linked diubiquitin, the applied shear stress was that of 33 s^−1^ for 90 h. (**c**) Fibril formation of sheared K63-linked diubiquitin (purple), linear diubiquitin (black) and hexaubiquitin (red), as followed by ThT fluorescence. K48-linked diubiquitin (orange) and monoubiquitin (green) did not show relevant increases in fluorescence. Shear stress was applied by agitation at a rotational speed of 25 s^−1^. (**d**) Comparative analysis of the kinetics of fibril formation by polyubiquitin chains of different linkage type and chain length with transition temperature. Rate constants were obtained by fitting the data to first-order kinetics. The values represent the average of two independent experiments. Error bars, the standard error of the mean. *P<0.05 (Student’s *t*-test).

We also found that not only heat but also mechanical stress induces the formation of amyloid-like fibrils of polyubiquitin chains. ThT fluorescence observations indicated that polyubiquitin chains formed ThT-positive aggregates in response to moderate agitation at a rotational speed as low as 33 s^−1^, regardless of chain length or linkage type, whereas monoubiquitin displayed no noticeable change under the same conditions (Fig. 4b and Supplementary Fig. 2b). EM images showed that the aggregates formed by mechanical stress are made of fibrils similar to those of amyloid-like fibrils (Fig. 4a).

**Figure 5:**
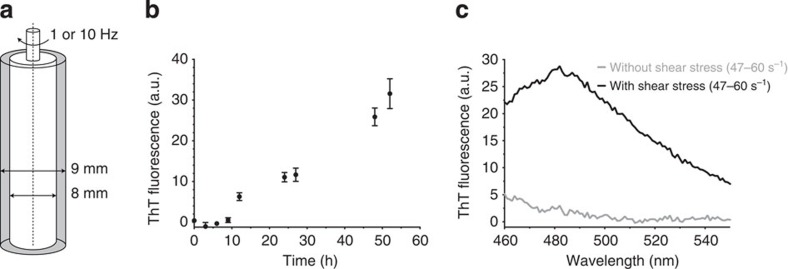
Quantitative shear stress analysis in a Couette cell. (**a**) Schematic diagram of the rotational flow device used. (**b**) Fibril formation of linear hexaubiquitin in response to shear rates of 470–600 s^−1^ in a Couette cell. Error bars, the standard error of the mean. (**c**) Thioflavin T fluorescence emission spectra of non-treated linear hexaubiquitin (grey) and linear hexaubiquitin sheared in a Couette cell at a rate of 47–60 s^−1^ for 72 h (black).

Next, we measured the rate of fibril formation using a ThT fluorescence assay at a rotational speed of 25 s^−1^ to compare the influence of these hydrodynamic forces on different types of ubiquitin. The order of fibril formation tendency by shear stress was the same as that observed for the transition temperature of the polyubiquitin chains, whereby linear chains aggregated at the lowest temperature, followed by K63- and then K48-linked chains (Fig. 4c,d). Quantitative analysis using controlled shear stress in a Couette cell indicated that hexaubiquitin formed fibrils in response to a shear rate of 47–60 s^−1^ (Fig. 5c), which is of the same order as that required to induce the aggregation of other fibrillogenic proteins, such as Aβ, insulin and β-lactoglobulin, in a Couette cell^14^. These results suggest that the longer the ubiquitin chains, the more easily they form fibrillar aggregates under conditions close to physiological conditions. This tendency may be caused by the increase in molecular anisotropy in polyubiquitin chains; that is, elongated molecules undergo larger anisotropic Brownian motions, and thus may be more easily affected by external mechanical stress and temperature.

### Ubiquitylated proteins also form fibrils by heat or shearing

**Figure 6:**
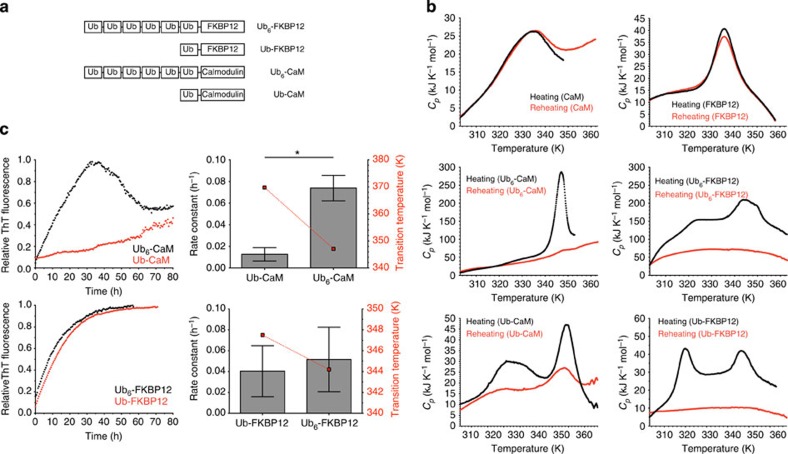
Ubiquitylation impairs the folding reversibility of proteins and induces formation of amyloid-like fibrils. (**a**) Schematic diagram of ubiquitylated proteins. (**b**) Differential scanning calorimetry (DSC) traces of calmodulin (CaM) (left upper) and FKBP12 (right upper), showing that the thermal unfolding of these proteins is reversible. DSC traces of Ub_6_-CaM (left middle), Ub_6_-FKBP12 (right middle), Ub-CaM (left lower) and Ub-FKBP12 (right lower), indicating that the thermal unfolding of ubiquitylated proteins is irreversible. Black traces represent initial heating and red traces show reheating. (**c**) Fibril formation of sheared Ub-CaM (left upper, red), Ub_6_-CaM (left upper, black), Ub-FKBP12 (left lower, red) and Ub_6_-FKBP12 (left lower, black) as followed by ThT fluorescence. Comparative analysis of the fibril formation kinetics of ubiquitylated CaM (right upper) and FKBP12 (right lower) with transition temperature. Shear stress was applied as an agitation at a rotational speed of 25 s^−1^. Rate constants were obtained by fitting the data to first-order kinetics. The values represent the average of two independent experiments. Error bars, the standard error of the mean. *P<0.05 (Student’s *t*-test).

Next, we found that ubiquitylated proteins also form amyloid-like aggregates under moderate conditions. As models for substrate proteins, we chose rat calmodulin and human FK506-binding protein (FKBP12), both of which are ubiquitylated *in vivo*^15^,^16^, and constructed their ubiquitylated forms in which monoubiquitin or linear hexaubiquitin was attached to the amino group of the N-terminal residue of the protein (that is, Ub-calmodulin, Ub_6_-calmodulin, Ub-FKBP12 and Ub_6_-FKBP12) (Fig. 6a). The DSC thermographs of ubiquitylated calmodulin and FKBP12 displayed irreversible thermo-transitions similar to those of ubiquitin chains, and aggregates were obtained not only by heat but also by shear stress (Fig. 6b). Although EM observations did not clearly reveal whether the aggregates contain fibrils, the CD spectra and ThT binding of the aggregates resembled to those of fibrils formed by polyubiquitin chains (Supplementary Fig. 3a,b). In contrast, non-ubiquitylated substrate proteins displayed reversible thermo-transitions and no aggregates were observed (Fig. 6b). Calmodulin conjugated to a longer polyubiquitin chain formed fibrils more easily than that conjugated to monoubiquitin (Fig. 6c, upper right). Such effect was limited in the case of FKBP12 (Fig. 6c, lower right). Taken together, these results suggest that ubiquitylation has an effect on aggregation propensity and thermal reversibility of a substrate protein and that conjugation of longer ubiquitin chains appears to enhance aggregate formation even though such effect was limited in the case of FKBP12: the effect of ubiquitin chains on fibril formation might depend on the attached substrate protein.

### Autophagy specifically degraded polyubiquitin aggregates

**Figure 7:**
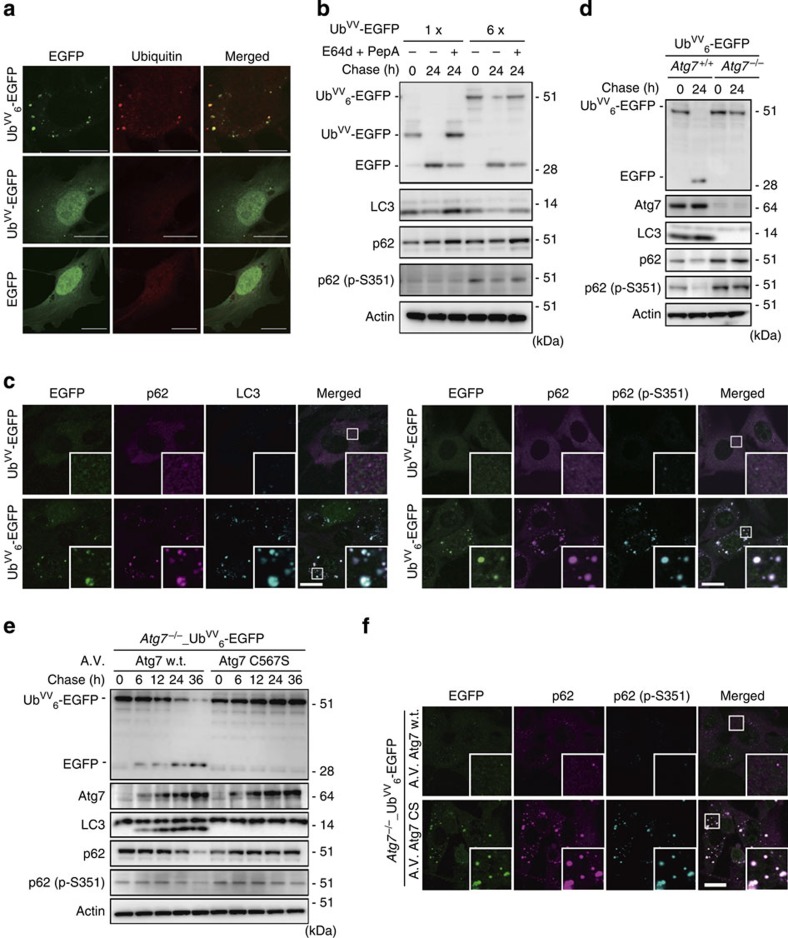
*In vivo* aggregation of polyubiquitin chains and degradation by macroautophagy. (**a**) MEFs were transiently transfected with Ub^VV^_6_-EGFP (top), Ub^VV^-EGFP (middle) or EGFP (bottom) and imaged 48 h after transfection. No ubiquitin-positive aggregates were detected in cells expressing Ub^VV^-EGFP and EGFP, whereas ubiquitin-positive aggregates of up to 2 μm in diameter were observed in cells expressing Ub^VV^_6_-EGFP. Scale bar, 20 μm. Data are representative of three independent experiments. (**b**) Immortalized wild-type MEFs harbouring two regulator gene cassettes, CAG-rtTA and either TRE-Ub^VV^_6_-EGFP or TRE-Ub^VV^-EGFP, were cultured for 24 h in the presence of Dox to induce expression of Ub^VV^_6_-EGFP or Ub^VV^-EGFP. Subsequently, the cells were cultured in the absence of Dox for 24 h. E64d and pepstatin were added as indicated. The cell lysates were prepared and immunoblotted with the indicated antibodies. Data are representative of two independent experiments. (**c**) The MEFs shown in **b** were cultured for 24 h in the presence of Dox to induce expression of Ub^VV^_6_-EGFP or Ub^VV^-EGFP, and then immunostained with p62 and LC3 antibodies or p62 and Ser351-phosphorylated p62 antibodies. Scale bar, 20 μm. Data are representative of three independent experiments. (**d**) Immortalized wild-type and *Atg7*-deficient MEFs were stably transfected with two regulator gene cassettes, CAG-rtTA and TRE-Ub^VV^_6_-EGFP. The cells were cultured for 24 h in the presence of Dox to induce expression of Ub^VV^_6_-EGFP. Subsequently, the cells were cultured in the absence of Dox for 24 h. The cell lysates were prepared and immunoblotted with the indicated antibodies. Data are representative of two independent experiments. (**e**) After induction of Ub^VV^_6_-EGFP in the *Atg7*-knockout MEFs as shown in **d**, Atg7 or Atg7 C567S was expressed using an adenovirus system. At the indicated time points, cell lysates were prepared and immunoblotted with the indicated antibodies. Data are representative of two independent experiments. (**f**) MEFs treated as shown in **e** were immunostained with p62 and Ser351-phosphorylated p62 antibodies, 36 h after infection with the indicated adenovirus vectors. Scale bar, 20 μm. Data are representative of three independent experiments. A.V., adenovirus; w.t., wild type.

We considered that the chain length-dependent aggregation observed *in vitro* might be related to the formation of ubiquitin-positive inclusion bodies in cells. To elucidate this possibility, we checked whether ubiquitylated proteins form aggregates intracellularly. We expressed EGFP attached to linear hexaubiquitin in which the C-terminal di-amino acids (G-G) of each ubiquitin unit were replaced by V-V (Ub^VV^_6_-EGFP) in mouse embryonic fibroblasts (MEFs). The cells displayed several EGFP-positive plaques of up to 2 μm in diameter (Fig. 7a, upper). In addition, expression of linear hexaubiquitin (Ub^VV^_6_) without any protein attached also resulted in the formation of intracellular insoluble aggregates (Supplementary Fig. 4). By contrast, in MEFs expressing monoubiquitin-attached EGFP (Ub^VV^-EGFP), the EGFP fluorescence was smoothly distributed across the cytosol and the nucleus, as observed in control cells expressing EGFP, except for the occasional formation of a few small spots in a small fraction of cells (Fig. 7a, middle, bottom and Supplementary Fig. 4). Thus, these results indicate that whether ubiquitin chains form intracellular aggregates or not is dependent on their chain length.

Clearance of ubiquitin-positive aggregates has been shown to depend on a type of selective autophagy, which degrades aggregated proteins (aggrephagy)^9^. In aggrephagy, ubiquitin on the aggregated structures leads to the assembly of ubiquitin-adaptor proteins such as p62 and NBR1, as well as core Atg proteins. First, to determine whether p62 and NBR1 have the ability to bind to the fibrillar form of polyubiquitin or not, we used a method of solution NMR^17^, in which the exchange process (binding and dissociation) is imprinted onto the transverse relaxation rates of the free UBA domain. Estimation of transverse relaxation rates (^15^N-*R*_2_) from heteronuclear single quantum coherence (HSQC) spectra of the respective free UBA domain and the UBA domain titrated with polyubiquitin fibrils indicated that both UBA domains interact with polyubiquitin fibrils. When the ^15^N-labelled NBR1 domain was titrated with non-labelled polyubiquitin fibrils, its average ^15^N-*R*_2_ increased by 5.32±1.53 s^−1^ (s.d.) in a residue-specific manner (Supplementary Fig. 5a). In the case of the UBA domain of p62, the increase was 2.94±1.10 s^−1^ (s.d.) (Supplementary Fig. 5b). Indeed, it has been shown that the interaction of amyloid-β monomers with the surface of amyloid-β protofibrils leads to an increase in ^15^N-*R*_2_ of the monomeric form by ~2 s^−1^, which is of the same order as our results^18^. We thus assume that both UBA domains interact with the surface of polyubiquitin fibrils, but it will be necessary to quantitatively probe the interaction kinetics in the near future.

Finally, we tested the possibility that the Ub^VV^_6_-EGFP aggregates serve as an initiation signal for aggrephagy. Autophagic flux assays revealed that expression of Ub^VV^_6_-EGFP did not affect the turnover of LC3-II, which represents autophagosome formation^19^, as did expression of Ub^VV^-EGFP (Fig. 7b). By contrast, degradation of Ser351-phosphorylated p62 in lysosomes, a hallmark of aggrephagy^20^, was induced by expression of Ub^VV^_6_-EGFP, but not Ub^VV^-EGFP (Fig. 7b). We also observed extensive co-localization of endogenous LC3 with aggregates positive for Ub^VV^_6_-EGFP (Fig. 7c). As expected^9^, p62 was also recruited to these structures and found to be phosphorylated at Ser351 (Fig. 7c). The loss of autophagy-related 7 (*Atg7*), an essential gene for autophagy, inhibited degradation of Ub^VV^_6_-EGFP, p62 and Ser351-phosphorylated p62 (Fig. 7d). Expression of wild-type Atg7, but not the active site mutant Atg7 C567S (ref. 21) restored degradation of these proteins (Fig. 7e). In accordance with these biochemical data, immunofluorescence staining revealed Ub^VV^_6_-EGFP aggregate structures positive for Ser351-phosphorylated p62 in MEFs expressing the Atg7 C567S mutant (Fig. 7f). Although soluble linear-ubiquitylated proteins are partly degraded by the ubiquitin–proteasome system^22^, our results indicate that aggrephagy mainly contributes to the clearance of insoluble ubiquitin-positive aggregates. In support of our data, we recently found that aggregates formed in hepatocytes with proteasome deficiency were selectively entrapped by autophagosomes, and pathological features of livers with impaired proteasome activity were exacerbated by simultaneous suppression of autophagy^23^. Remarkably, a highly sensitive polyubiquitin chain quantification method^24^ revealed that insoluble proteins from livers with impaired proteasome activity contained all linkage types of polyubiquitin chains (Supplementary Fig. 6b). Likewise, we found that insoluble polyubiquitin chains accumulating in autophagy-deficient livers showed no linkage specificity^25^ (Supplementary Fig. 6b). These *in vivo* analyses suggest that all types of polyubiquitin chains on proteins have the potential to induce their fibrillar aggregate formation followed by induction of autophagy, which serves a cytoprotective function.

## Discussion

In this manuscript, we describe a novel and unexpected nature of ubiquitin; that is, in contrast to the rigid structure of a single ubiquitin, ubiquitin becomes thermodynamically unstable when it is conjugated to another ubiquitin molecule or to another protein. This is unexpected because proteins with repeating identical domains have been considered to show increasing folding stability with an increasing number of domains^26^.

So why does length-dependent destabilization occur in polyubiquitin chains? One possibility is domain swapping, in which a secondary or tertiary element of a monomeric protein is replaced by the corresponding element of another protein molecule^27^. Recently, it has been reported that connecting of immunoglobulin domains in tandem causes fibril formation via a domain-swapping mechanism^28^,^29^. Intriguingly, a similar domain-swapping event has been proposed for diubiquitin^30^. Therefore, elongation of ubiquitin into a ubiquitin chain may promote the formation of both intra- and inter-molecular domain-swapped structures. This may explain the length-dependent thermodynamic destabilization that we observed in polyubiquitin chains.

The thermodynamic stability of monoubiquitin is exceptionally high. Even polyubiquitin chains may have a higher thermodynamic stability than other cytosolic proteins. However, the temperature at which long polyubiquitin chains form fibrils is comparable with that at which several other amyloid-prone proteins form fibrils: myoglobin (horse skeletal muscle) forms fibrils by heating to 338 K (ref. 31) and β-lactoglobulin does so by heating to 353 K (ref. 32). In addition, it is noteworthy that long polyubiquitin chains form insoluble fibrils at moderate shear stress under physiological temperature and pH (Fig. 5b,c), implying that ubiquitin fibrils may form under physiological conditions even though the experimental conditions did not exactly mimic *in vivo* situations. It is currently not well understood, in what capacity melting temperatures of proteins determined *in vitro* correlate with protein aggregation *in vivo*. As the intracellular environment markedly differs from the test tube system in which we determined these unfolding temperatures, it is plausible that additional factors trigger the aggregation of a given ubiquitin chain in living cells.

An involvement of ubiquitylation in the sequestration and degradation of misfolded proteins has been already described^33^,^34^ and it is reported that monoubiquitylation triggers sequestration of α-synuclein aggregates^35^. However, the mechanism of how ubiquitylation induces aggregation-associated sequestration has remained elusive. Our *in vitro* data suggest that ubiquitylation has an effect on aggregation propensity and thermal folding reversibility of a substrate protein (Fig. 6b), which could be connected to the sequestration and degradation of misfolded proteins by ubiquitylation. But, how monoubiquitylation causes protein aggregation and why the kinetics of fibril formation depends on a substrate protein have been elusive; therefore, further analysis is needed to clarify these critical issues. In addition, our *in vivo* data indicate that simple overexpression of EGFP attached to linear hexaubiquitin, but not monoubiquitin, was sufficient for the formation of aggregate structures in cells (Fig. 7a). This observation underscores that fibril formation occurs *in vivo*, when polyubiquitylated proteins, which accumulate owing to proteasome dysfunction or dysregulated deubiquitylation, are exposed to intracellular forces arising from cytoplasmic streaming, macromolecular crowding or (non-) specific protein interactions over a period of time. It will be necessary to determine the kind of intracellular forces involved and the length of time needed for ubiquitin fibril formation.

Although autophagy has been considered to be a non-selective bulk degradation system of the cell, mounting evidence is pointing to other autophagy modes that selectively degrade aggregated proteins (aggrephagy), damaged mitochondria (mitophagy) and invading bacterial cells (xenophagy)^36^. Thus, inactivation of autophagy leads to an accumulation of both cytoplasmic protein inclusions and excess deformed organelles, which causes liver injury, diabetes, heart disease and neurodegeneration^37^. Under the conditions of each processes, namely aggrephagy, mitophagy and xenophagy, different target proteins are ubiquitylated by distinct E3s such as Chip, Parkin and LRSAM1 (ref. 36), implying that polyubiquitin chains of varying topology give rise to different types of selective autophagy. Nevertheless, in any of these types of selective autophagy, ubiquitylation triggers a common transduction signal—namely, the assembly of core Atg proteins and adaptor proteins such as p62 around the autophagic cargo. Therefore, our observation that the formation of fibrils by polyubiquitin chains was linkage-type independent (Fig. 1d) suggests that the ubiquitin fibril plays a critical role in selective autophagy. We propose that polyubiquitin chains have two distinct biological roles: one is a linkage-specific signal for proteasome-mediated degradation and other non-proteolytic pathways. The other role is a linkage-independent, but length-dependent inducer of fibrillar structures for selective clearance by autophagy. The latter pathway would be cytoprotective: such insolubilization would prevent any undesired activities of the substrate proteins before protein aggregates start to accumulate in cells^38^,^39^.

In healthy cells, protein aggregates sequestered by ubiquitylation are degraded by selective autophagy before they form large inclusions (Fig. 7b). On the other hand, the activity of the ubiquitin–proteasome system decreases with aging^40^ and loss (that is, dysfunction or inactivation) of autophagy has been previously described in senescent cells^41^,^42^. Accordingly, most elderly patients suffering from neurodegenerative diseases may have insufficient activity of autophagy—namely the capacity to specifically degrade intracellular ubiquitin-positive aggregates (ubiquitin fibrils). As a result, aging- and/or disease-associated inactivation of the proteasome and/or autophagy pathways may result in cytotoxic accumulation of ubiquitin-positive aggregates, even in the absence of aggregate-prone proteins related to conformational diseases. Ultimately, such ubiquitin-positive aggregates may be sequestered into so-called inclusion bodies, particularly in non-dividing cells such as neurons and myocytes. We propose that this is why most inclusion bodies observed in neurodegenerative diseases that have been reported contain ubiquitin as a major constituent^7^. A unique feature of polyubiquitin chains is that it is the wild-type protein that forms fibrils; this feature is in stark contrast to other amyloid-forming proteins, many of which are truncated or carry mutations such as Aβ or SOD1 (refs 12, 43). Therefore, our proposed model would take into account the pathological hallmarks of human sporadic proteinopathies without genetic mutations.

Collectively, intracellular ubiquitylation not only aids recruitment of proteins to the proteasome in solution, but also shields the cell from undesired activities of substrate proteins by encapsulating them in solid aggregates. Last, the structure of the ubiquitin fibrils acts as an initiation signal for autophagy. To our knowledge, this paper is the first to report that ubiquitin chains function as a driving force to form fibrillar aggregates that have chain-length dependency but no linkage specificity and that these aggregates, whose accumulation disturbs cellular homeostasis, can act as direct substrates for removal by selective autophagy. The two biological roles of polyubiquitin chains (that is, a canonical linkage-specific signal and an inducer of fibril formation) may function independently and, accordingly, future studies should aim to elucidate the underlying mechanism that discriminates between them in a spatiotemporal fashion.

## Methods

### Protein preparation

Mouse E1 enzyme UBA1 was expressed in Sf9 cells, yeast K63-linked E2 enzyme Ubc13-Mms2 complex was expressed in *Escherichia coli* strain BL21 Codon plus (*DE3*) RIL and all other recombinant proteins were expressed in *E. coli* strain BL21 (*DE3*). Untagged human ubiquitin was purified by ion exchange and size-exclusion chromatography^44^. Human 12-kDa FK506-binding protein (FKBP12) was expressed as a fusion protein with an N-terminal glutathione *S*-transferase (GST) and small ubiquitin-like modifier (SUMO)-1 protein tag. After cleavage of the protein tag by GST-SENP2 protease, FKBP12 was further purified by ion exchange and size-exclusion chromatography^45^. Untagged rat calmodulin was purified by Phenyl Sepharose Fast Flow (GE Healthcare) and size-exclusion chromatography^46^. N-terminal hexa-histidine (His_6_) tagged mouse UBA1, K48-linked E2 enzyme E2-25K and linear E2 enzyme UbcH7 was purified by Ni-NTA affinity chromatography^22^,^44^,^47^. Yeast Ubc13 was co-expressed with N-terminal His_6_-tagged yeast Mms2, followed by purification by Ni-NTA affinity and size-exclusion chromatography^44^. Human HOIL-1L (1–191) was co-expressed with N-terminal His_6_-tagged human HOIP (476–1,072) and the HOIL-1L-HOIP complex (human linear E3 ligase) was purified by Ni-NTA affinity chromatography^22^. K48-linked and K63-linked polyubiquitin chains were enzymatically synthesized by the appropriate E1, E2 and E3 enzymes shown above^47^. Linear polyubiquitin chains were both expressed recombinantly and synthesized enzymatically^22^,^47^. Separation of cyclic and non-cyclic K48-linked polyubiquitin chains has not been performed. Met1-monoubiquitylated and hexaubiquitylated FKBP12, which contain an additional DGGS sequence between ubiquitin and FKBP12, and an HRV3C-cleavable C-terminal His_6_-tag, were expressed and purified by Ni-NTA affinity chromatography. After cleavage of the C-terminal His_6_-tag by HRV3C protease, the fusion proteins were further purified by ion exchange and size-exclusion chromatography. Met1-mono- and hexaubiquitylated calmodulin containing an HRV3C-cleavable C-terminal His_6_-tag were expressed and purified in the same manner. Calcium ions, to which calmodulin and ubiquitylated calmodulin bind with high affinity, were removed by EGTA during the purification.

### Differential scanning calorimetry

Thermal denaturation curves were acquired by a Nano DSC instrument (TA Instruments Inc.). The scan rate was 1 K min^−1^, and protein concentrations ranged from 0.2 to 1 mg ml^−1^. The buffer used in the calorimetric experiments of (poly-)ubiquitin and (Met1-ubiquitylated-)calmodulin was phosphate-buffered saline (PBS; 137 mM NaCl, 8.1 mM Na_2_HPO_4_, 2.68 mM KCl, 1.47 mM KH_2_PO_4,_ pH 7.4). In the case of (Met1-ubiquitylated-)FKBP12, 0.5 mM TCEP was included to prevent cysteine-mediated dimerization. Reheating experiments were performed in the same manner after heating until the peak finished and mild cooling to room temperature. Analysis was performed using CpCalc (TA Instruments Inc.) and data were reported as heat capacity (kJ K^−1^ mol^−1^). The transition temperature was defined as the temperature corresponding to the peak top. However, precise thermodynamic parameters could not be acquired owing to the irreversibility of the melting reaction *in vitro*.

### Transmission electron microscopy

TEM images were obtained using a JEM-1011 transmission electron microscope (JEOL). All the samples were diluted to 100 μg ml^−1^ in 25 mM Tris-HCl pH 8 and 150 mM NaCl, loaded onto a carbon grid and negatively stained with 2% (w/v) uranyl acetate or PTA (phosphotungstic acid). Scale bars were estimated by measuring tobacco mosaic virus under identical conditions. Images were analysed via ImageJ 1.45s.

### Fluorescence spectroscopy

Fluorescence was quantified on a FluoroMax4 (HORIBA) spectrometer at 298 K by excitation at 440 nm with acquisition of emission spectra over wavelengths of 460 to 550 nm with the slit width set at 5 nm. Monomeric samples were diluted to a final concentration of 5.8 μM in analysis buffer containing 25 mM Tris-HCl pH 8, 150 mM NaCl and 25 μM thioflavin T. Polyubiquitin chains and ubiquitylated proteins were diluted to an equimolar concentration (5.8 μM) of monomeric ubiquitin subunits. In the case of (Met1-ubiquitylated-)FKBP12, 0.5 mM TCEP was included. The spectral contribution of the buffer was subtracted from the acquired spectra. To monitor fibril growth, the ThT fluorescence intensity at 480 nm was measured every 30 min with continuous stirring at 25 s^−1^ by a stirrer bar (Bel-Art Products). The fluorescence intensities at time *t* after the start, I(*t*), were fitted to the equation I(*t*)=I(*t*_*∞*_) [1−exp (−*kt*)] to obtain the rate constant *k*.

### Circular dichroism spectroscopy

Circular dichroism spectra were collected on a J-820 spectrophotometer (JASCO) from 250 to 200 nm in 0.1-nm intervals. Data were measured at 298 K with a 1-mm path length in 25 mM Tris-HCl pH 8, 150 mM NaCl, containing 0.5 mM TCEP in the case of (Met1-ubiquitylated-)FKBP12. The concentration of all proteins used in the measurements was adjusted to 0.2 mg ml^−1^. Data were reported as mean residue ellipticities. The spectral contributions by the buffer were subtracted.

### Shear stress in a Couette cell

Controlled shear stress^48^ was produced by an SM-101 instrument (As One), which incorporated an iron inner cylinder (8 mm) in a custom-built quartz cell (9 mm) at room temperature (298 K; Fig. 5a). Samples treated with shear stress were collected from the shear cell at each time point. Linear hexaubiquitin was diluted to an equimolar concentration (116 μM) of monomeric ubiquitin subunits in analysis buffer containing 25 mM Tris-HCl pH 8 and 150 mM NaCl. The samples were exposed to a moderate shear rate of 47–60 or 470–600 s^−1^. The shear rate in the Couette cell is calculated as 
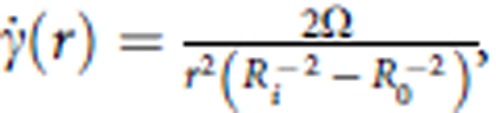
, where Ω is the angular frequency of the rotor (rad s^−1^), *R*_*i*_ is the radius of the rotor and *R*_0_ represents the radius of the quartz cell^49^,^50^.

### Transfection

MEFs were maintained in DMEM (Gibco) with 10% fetal bovine serum, penicillin (100 U ml^−1^) and streptomycin (100 μg ml^−1^). The cells were detached from the dish with 0.025% trypsin, and 2 × 10^6^ cells were centrifuged at 100 *g* for 10 min. The cell pellet was gently suspended in 100 μl of MEF Nucleofector solution (Amaxa) with 10 μg of expression plasmid pcDNA 3.1 cloned with EGFP, Ub^VV^-EGFP or Ub^VV^_6_-EGFP, which contain an additional MASH sequence in front of ubiquitin and an additional GGSG sequence between ubiquitin and EGFP. Ub^VV^ indicates a C-terminal Val-Val ubiquitin mutant with the sequence MQIFVKTLTGKTITLEVESSDTIDNVKAKIQDKEGIPPDQQRLIFAGKQLEDGRTLADYNIQKESTLHLVLRLRVV. The cell suspension was electroporated by using the T-020 program (Amaxa).

### Immunoblot analysis

Samples were separated using 12% NuPAGE Bis-Tris gels (Invitrogen) in MOPS-SDS buffer, followed by transferring to polyvinylidene difluoride membranes. Antibodies against ubiquitin (Santa Cruz Biotechnology, Inc., P4D1), p62 (Progen Biotechnik, GP62-C), LC3B (Cell Signaling Technology, #2775), GFP (Invitrogen) and Actin (Chemicon International, Inc., MAB1501R) were purchased from the indicated suppliers. Anti-phosphorylated p62 polyclonal antibody was raised in rabbits using the peptide Cys+KEVDP(pS)TGELQSL as an antigen^20^. The polyclonal antibody against Atg7 was raised in rabbits using the synthetic peptide VVAPGDSTRDRTLDQQ, which corresponds to the amino acid residues 556–571 as an antigen^21^. For blotting, indicated antibodies were used at a dilution of 1:500. Uncropped blots are shown in Supplementary Fig. 8.

### Immunocytochemistry

Cells grown on coverslips were fixed with 4% paraformaldehyde in PBS and permeabilized with 0.1% Triton X-100 or 50 μg ml^−1^ digitonin in PBS. After washing with PBS, the coverslips were blocked with PBS containing 10% normal goat serum (Jackson Immuno Research) for 1 h or 0.1% (w/v) gelatin (Sigma-Aldrich) in PBS for 30 min, and then incubated overnight with 150 or 200-fold diluted solution of primary antibodies against ubiquitin (Dako, Z 0458), p62 (Progen Biotechnik, GP62-C), Ser351-phosphorylated p62 and/or LC3B (Cell Signaling Technology, #2775). After washing with PBS, the coverslips were incubated with a 1,000-fold diluted solution of Alexa Fluor-conjugated goat anti-guinea pig and/or anti-rabbit IgG secondary antibodies (Invitrogen) for 1 h. Images were taken by confocal laser scanning microscopy using an FV1000 microscope (Olympus). Z-projection stack images were acquired with z-steps of 0.5 μm. Image contrast and brightness were adjusted using Photoshop CS4 (Adobe Systems, Inc.).

## Author contributions

D.M. and M.S. designed the experiments. D.M. and E.W. conducted most experiments. H.F. performed DSC analysis. M.H. assisted with fluorescence and CD spectroscopy. T.F. and K.N. contributed to electron microscopy experiments. Y.-S.S. and S.K performed the cell biological studies. H.T. and Y.S. performed mass spectroscopic analysis. K.A., M.A., H.T. and K.I. supervised the project. M.K. and K.T. coordinated the research. D.M., E.W., K.T., M.K. and M.S. wrote the manuscript. All the authors discussed the results and commented on the manuscript.

## Additional information

**How to cite this article:** Morimoto, D. *et al*. The unexpected role of polyubiquitin chains in the formation of fibrillar aggregates. *Nat. Commun.* 6:6116 doi: 10.1038/ncomms7116 (2015).

## Supplementary Material

Supplementary Information Supplementary Figures 1-8, Supplementary Table 1, Supplementary Methods and Supplementary References
