# Supplementary material for: The unexpected role of polyubiquitin chains in the formation of fibrillar aggregates
Source: Nat Commun. 2015 Jan 20;6:6116. doi: 10.1038/ncomms7116 (PMC4309437; doi:10.1038/ncomms7116)
Supplement: Supplementary Information — Supplementary Figures 1-8, Supplementary Table 1, Supplementary Methods and Supplementary References [file ncomms7116-s1.pdf]

Supplementary Figures

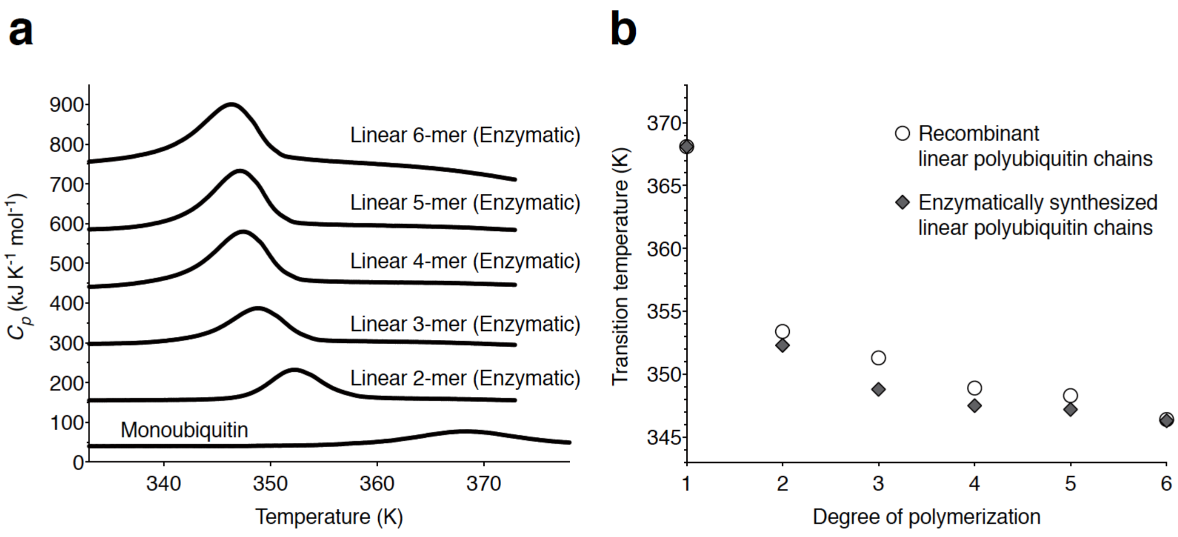

Supplementary Figure 1. Comparative analysis of thermal denaturation of enzymatically synthesized polyubiquitin chains of different length. a, Differential scanning calorimetry traces of monoubiquitin and enzymatically synthesized linear polyubiquitin chains with up to six ubiquitin units. **b**, Transition temperatures are plotted against chain length for recombinant and enzymatically synthesized linear ubiquitin chains.

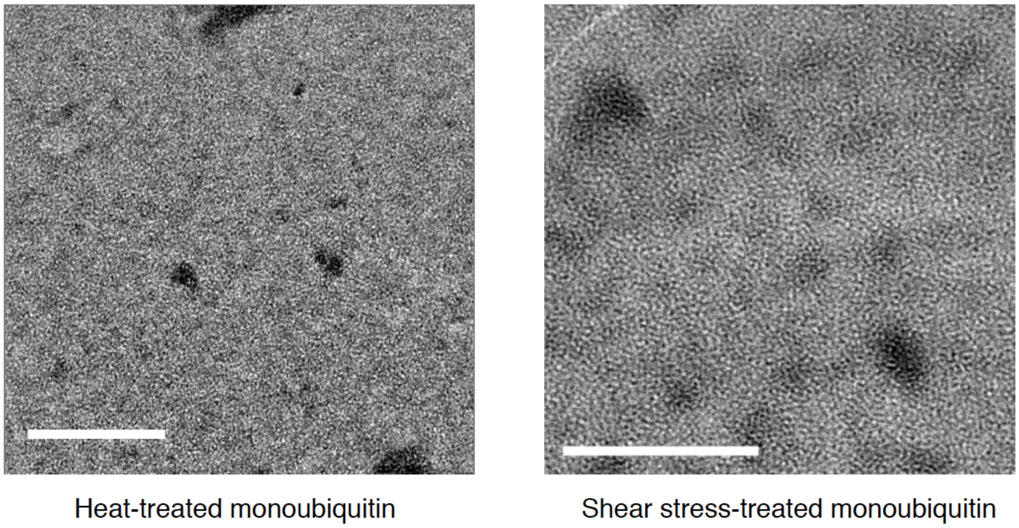

Supplementary Figure 2. Neither heat nor shear stress induces fibril formation of monoubiquitin . Electron Microscopy (EM) images of heat-treated monoubiquitin (**a**) and shear stress-treated monoubiquitin (**b**). Scale bars, 100 nm.

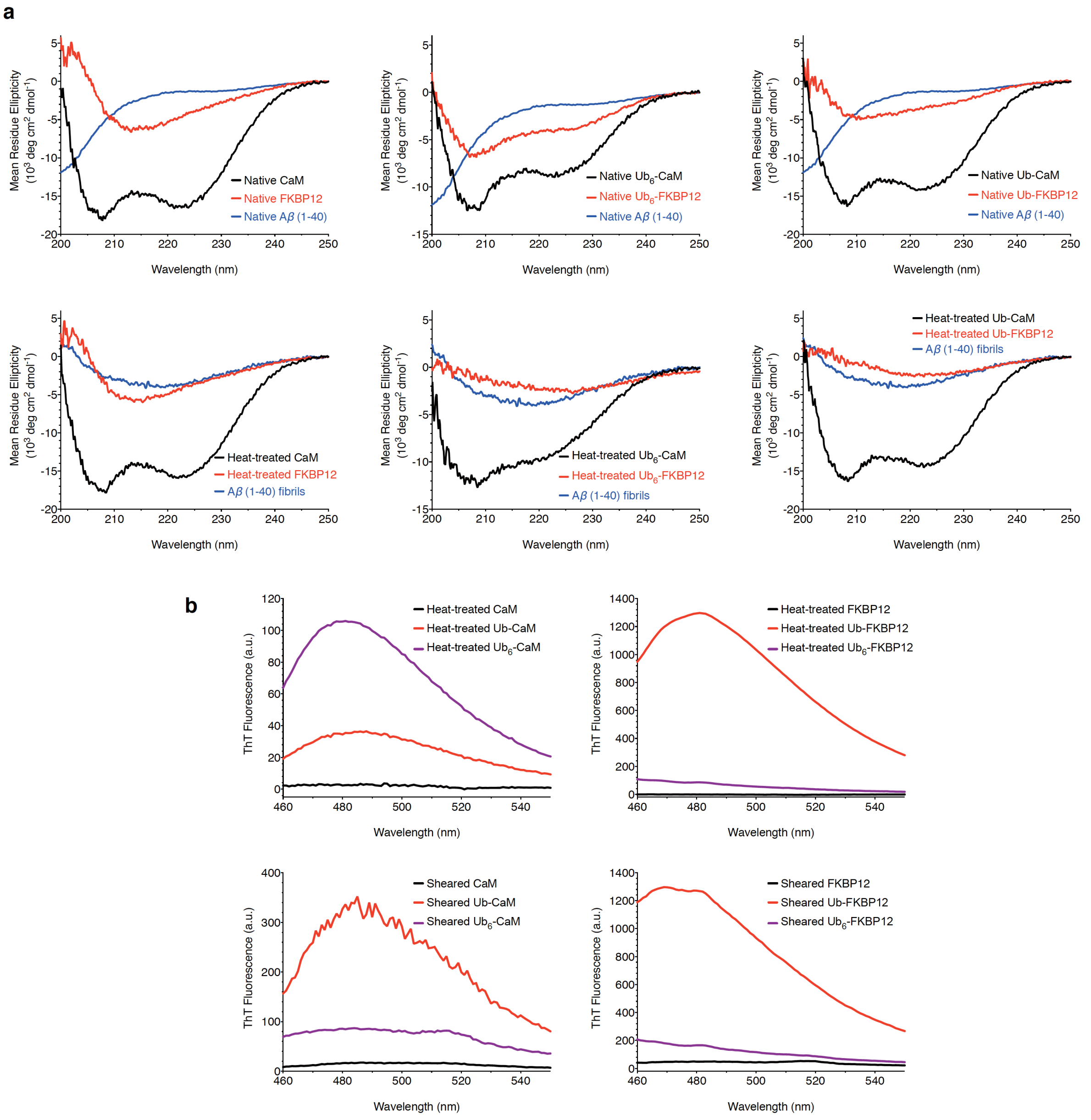

Supplementary Figure 3. Heat-induced secondary structural changes and thioflavin T fluorescence emission of ubiquitylated proteins. a, Circular dichroism spectra of native (upper) and heated (lower) CaM (left, black), FKBP12 (left, red), Ub_6-CaM (middle, black), Ub_6-FKBP12 (middle, red), Ub-CaM (right, black) and Ub-FKBP12 (right, red). For comparison, spectra of native Aβ (1-40) or Aβ (1-40) fibrils (blue) are shown in each spectrum. **b**, Thioflavin T fluorescence emission spectra of heated (upper) and sheared (lower) CaM (left, black), Ub-

CaM (left, red), Ub_6-CaM (left, purple), FKBP12 (right, black), Ub-FKBP12 (right, red) and Ub_6-FKBP12 (right, purple). Shear stress was applied as an agitation at a rotational speed of 25 s^{-1} for 90 hours.

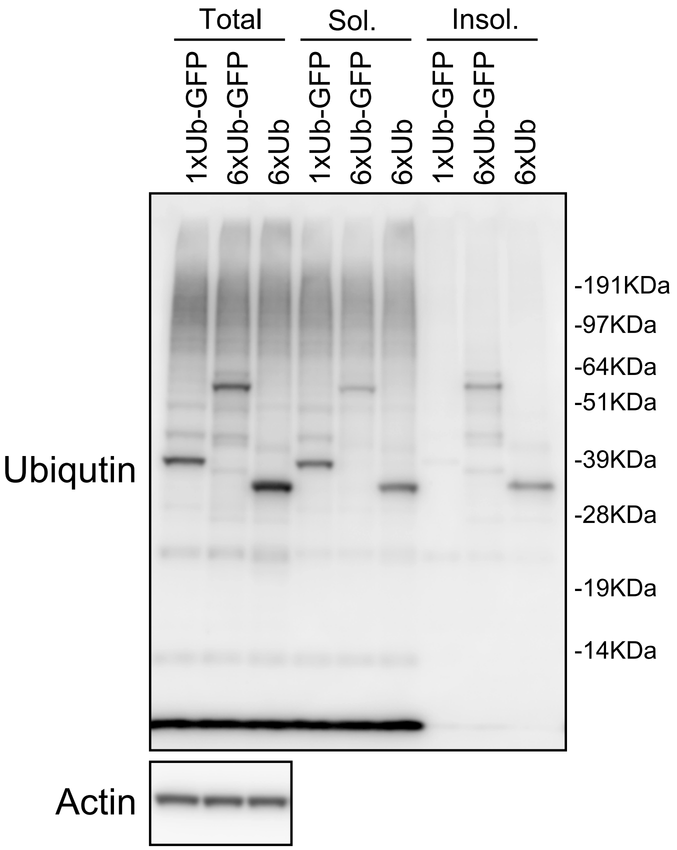

Supplementary Figure 4. Aggregate formation of polyubiquitin chains in cells is independent of conjugation to other proteins. HeLa cells were transiently transfected with pcDNA 3.1 (+) vectors encoding Ub^{VV}-EGFP (left), Ub^{VV}_6-EGFP (middle) and Ub^{VV}_6 (right). Total, soluble (Sol.), and insoluble (Insol.) fractions were subjected to immunoblotting with the indicated antibodies. Data are representative of two independent experiments.

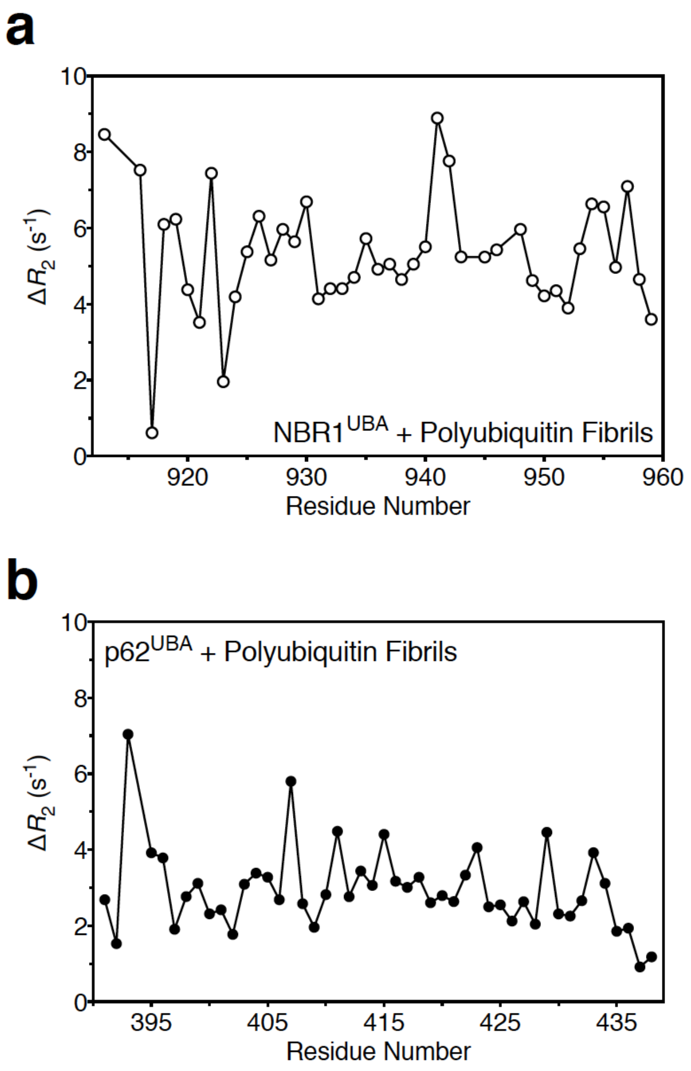

Supplementary Figure 5. Interaction of the UBA domains of p62 and NBR1 with polyubiquitin fibrils. Estimation of ΔR_2, the difference of the transverse relaxation rates (^{15}N-R_2) of free UBA domains and UBA domains titrated with polyubiquitin fibrils, at each residue position[1]. 200 μM of ^{15}N-labeled UBA domains of p62 (**a**) or NBR1 (**b**) were titrated with four fold molar excess of non-labeled polyubiquitin fibrils formed by heat-denaturation of linear hexaubiquitin V-V mutants. The concentration of polyubiquitin chains was defined as the concentration of monomeric ubiquitin subunits in a polymer. ^{15}N-R_2 values were obtained from

the full-width at half height (FWHH) of each peak in heteronuclear single quantum coherence (HSQC) spectra using nmrPipe[2]. Resonance assignments of the UBA domains were based on our previous works[3,4].

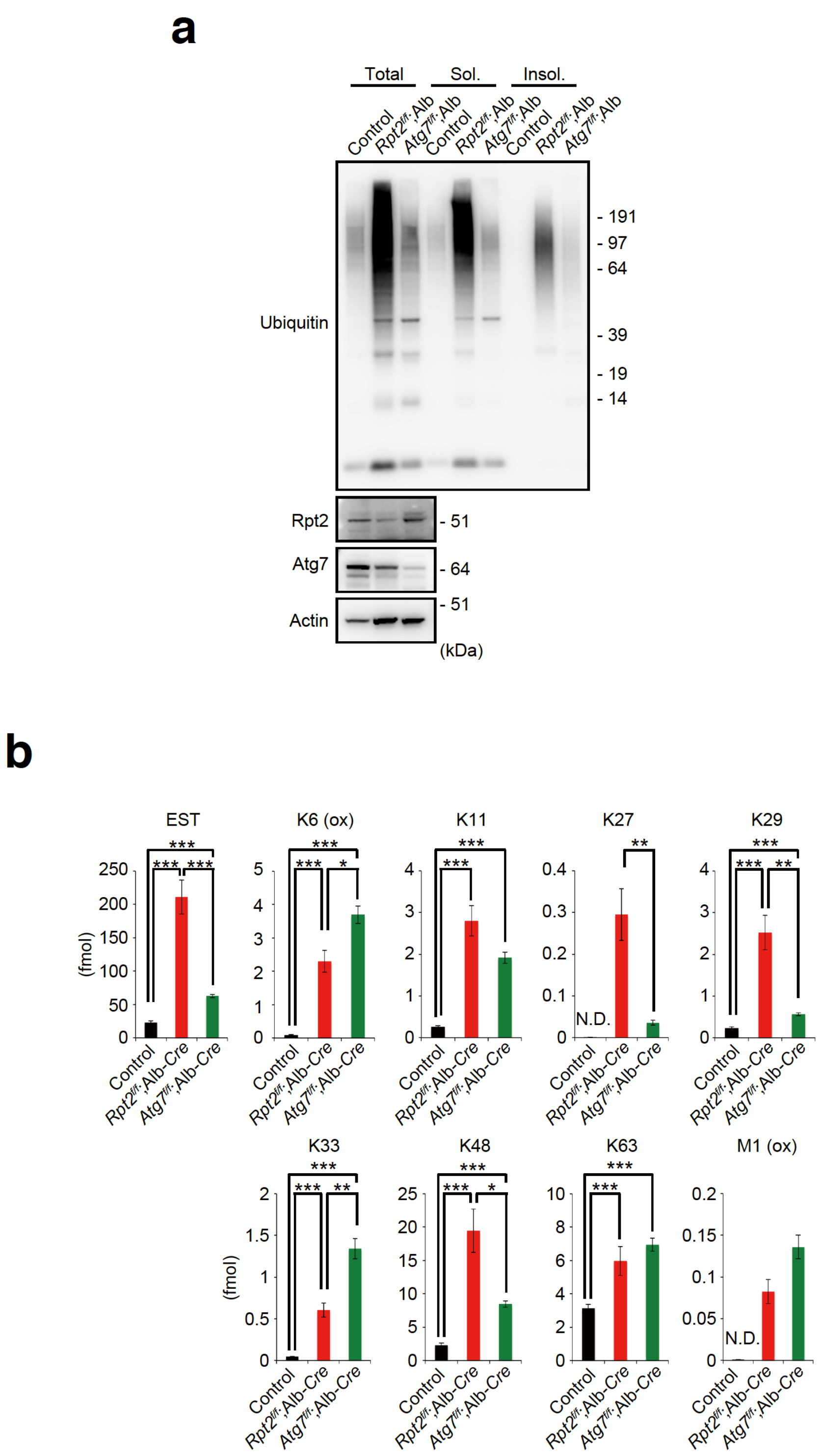

Supplementary Figure 6. Characterization of ubiquitylated aggregates in *Rpt2*$^{flox/flox}$;Alb-*Cre* and *Atg7*$^{flox/flox}$;Alb-*Cre* livers. (**a**) Liver homogenates were prepared from *Rpt2*$^{flox/flox}$;Alb-*Cre* and *Atg7*$^{flox/flox}$;Alb-*Cre* mice at postnatal day 30. Total, soluble (Sol.), and insoluble (Insol.)

fractions were subjected to immunoblotting with the indicated antibodies. Data were obtained from three independent experiments. (**b**) Absolute quantitation of total polyubiquitin chains (EST) and of each type of polyubiquitin chains in the insoluble fractions of control (n = 4), *Rpt2*$^{flox/flox}$;Alb-*Cre* (n = 4) and *Atg7*$^{flox/flox}$;Alb-*Cre* (n = 5) livers. Error bars represent the standard error of the mean. *$P < 0.05$, **$P < 0.01$, and ***$P < 0.001$ (Student's *t*-test).

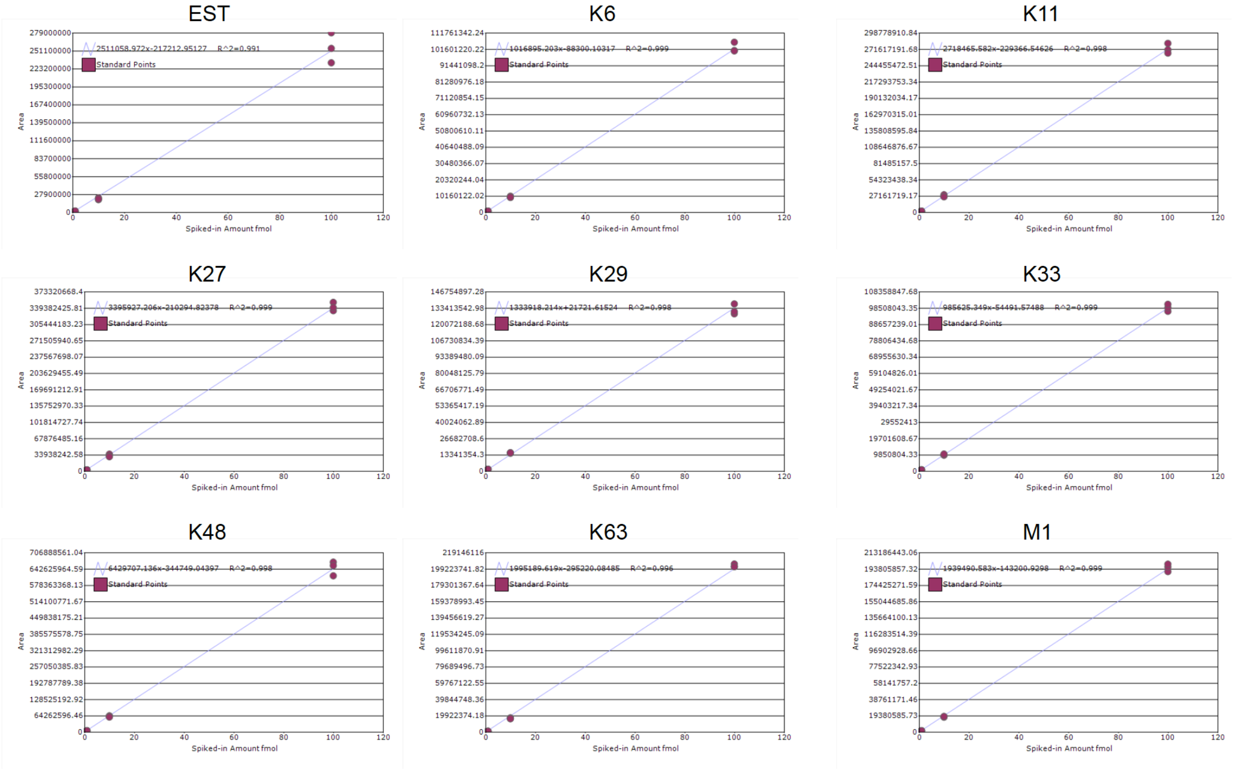

Supplementary Figure 7. Establishment of standard curves for ubiquitin peptides. All nine ubiquitin peptides, including eight ubiquitin linkages and ESTLHVLR (EST), were spiked into the *E. coli* matrix (250 ng on column, MassPREP, Waters) and analyzed by PRM in triplicate. Standard curves were established covering the range from 100 amol to 100 fmol in the *E. coli* matrix. The calculated standard curve regression lines for ubiquitin-linkage peptides are presented (R^2 = 0.991–0.999). The acceptable range of variation was set by the FDA guidelines for the lower limit of quantification (± 20%).

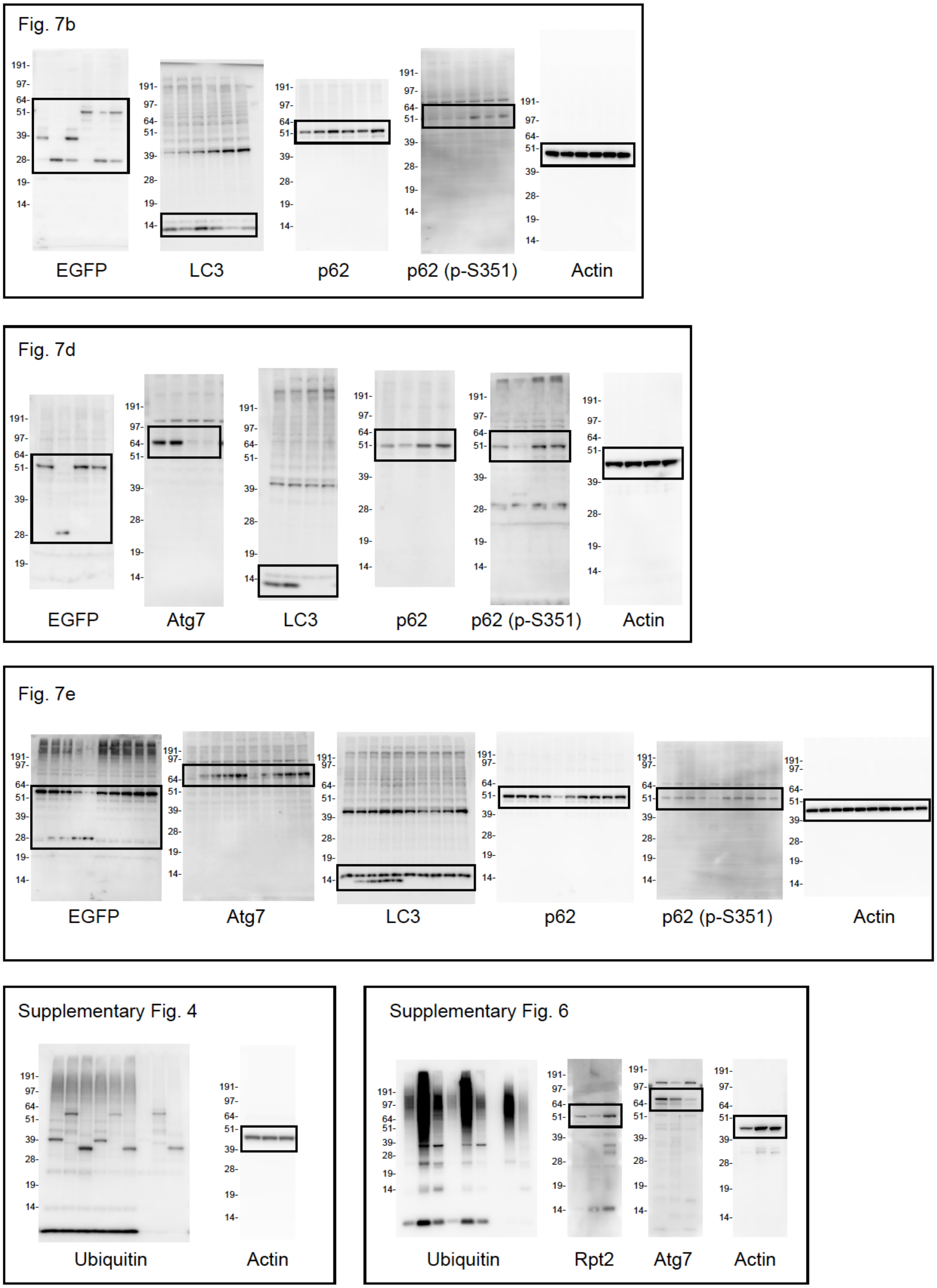

Supplementary Figure 8. Uncropped scans of immunoblots.

Supplementary Table

Supplementary Table 1. List of the ubiquitin peptides used in PRM analysis. In the peptide sequences, the following abbreviations are used: EST, K6, K11, K27, K29, K33, K48, K63, and M1 for the corresponding ubiquitin peptides; Heavy for isotopically labeled ($^{13}C^{15}N$) amino acids (e.g., "HeavyL" denotes isotopically labeled leucine); di-GlyGly for the ubiquitylation site; and Oxid for Met oxidation. For each peptide, the optimal precursor ions, product ions, and retention times are listed.

Abbreviation	Peptide Sequence	Precursor m/z (charge state)	Product ions for PRM	RT min
EST	ESTLHLVLR	534.314 (+2)	y3, y4, y5, y6, y7	41.69
	ESTLHLVL[HeavyL]R	537.83 (+2)		
K6 (ox)	M[Oxid]QIFVK[di-GlyGly]TLTGK	465.927 (+3)	y5, y6, y7	41.96
	M[Oxid]QIFVK[di-GlyGly]TL[HeavyL]TGK	468.266 (+3)		
K11	TLTGK[di-GlyGly]TITLEVEPSDTIENVK	801.427 (+3)	y8, y9, y10, y11, y12, y13	59.81
	TLTGK[di-GlyGly]TITLEVEPSDTIENV[HeavyV]K	803.431 (+3)		
K27	TITLEVEPSDTIENVK[di-GlyGly]AK	701.039(+3)	y6, y10, y11, y12	54.18
	TITLEVEPSDTIENV[HeavyV]K[di-GlyGly]AK	703.044 (+3)		
K29	AK[di-GlyGly]IQDK	408.732(+2)	y3, y4, y5	23.34
	AK[di-GlyGly]I[HeavyI]QDK	412.241(+2)		
K33	IQDK[di-GlyGly]EGIPPDQQR	546.613 (+3)	y3, y4, y6	28.72
	IQDK[di-GlyGly]EGIPP[HeavyP]DQQR	548.618 (+3)		
K48	LIFAGK[di-GlyGly]QLEDGR	487.6 (+3)	y4, y5, y6, y7, y8, y9	43.53
	LIFAGK[di-GlyGly]QL[HeavyL]EDGR	489.9391 (+3)		
K63	TLSDYNIQK[di-GlyGly]ESTLHLVLR	748.738 (+3)	y5, y6, y7, y8, y9, y10	62.23
	TLSDYNIQK[di-GlyGly]ESTLHLVL[HeavyL]R	751.077 (+3)		
M1 (ox)	GGM[Oxid]QIFVK	448.2389 (+2)	y3, y4, y5, y6	32.78
	GGM[Oxid]QIFV[HeavyV]K	451.2458 (+2)		

Supplementary Methods

Mice. *Rpt2*$^{f/f}$ mice[5] and *Atg7*$^{f/f}$ mice[6] were crossbred with Albumin-*Cre* transgenic mice[7] to generate *Rpt2*$^{f/f}$;Alb-*Cre* and *Atg7*$^{f/f}$;Alb-*Cre* mice, respectively. Mice were housed in specific pathogen–free facilities, and the Ethics Review Committee for Animal Experimentation of the Tokyo Metropolitan Institute of Medical Science approved the experimental protocols.

Quantitation of ubiquitin chains by mass spectrometry

Ubiquitin chains were quantitated by parallel reaction monitoring (PRM), a MS-based high-resolution quantification method[8]. In brief, insoluble proteins (20 μg) prepared as above were separated by SDS-PAGE on 4–12% NuPAGE Bis-Tris gels (Life Technologies) with a short run (3 cm). The gel region corresponding to a molecular weight of above 62 kDa was excised, diced into 1-mm^3 pieces, and subjected to in-gel trypsinization. Trypsinized peptides were extracted, spiked with nine ubiquitin AQUA peptides (K6-, K11-, K27-, K29-, K33-, K48-, K63-, and M1-linkages, and ESTLHVLR [EST]), and oxidized with 0.05% H_2O_2 in 0.1% trifluoroacetic acid at 4°C overnight. The peptides were analyzed in targeted MS/MS mode on a Q Exactive mass spectrometer coupled with an EASY-nLC 1000 liquid chromatograph (Thermo Fisher Scientific). Raw files were processed by PinPoint software version 1.3 (Thermo Fisher Scientific). The transition list and standard curves of the ubiquitin peptides are shown in Supplementary Table 1 and Supplementary Fig. 7, respectively.

Supplementary References

1. Fawzi, N. L., Ying, J., Torchia, D. A. & Clore, G. M. Probing exchange kinetics and atomic resolution dynamics in high-molecular-weight complexes using dark-state exchange saturation transfer NMR spectroscopy. *Nat Protoc* **7**, 1523-1533, (2012).

2. Delaglio, F. *et al*. NMRPipe: a multidimensional spectral processing system based on UNIX pipes. *J Biomol NMR* **6**, 277-293 (1995).

3. Isogai, S. *et al*. Crystal structure of the ubiquitin-associated (UBA) domain of p62 and its interaction with ubiquitin. *J Biol Chem* **286**, 31864-31874 (2011).

4. Walinda, E. *et al*. Solution Structure of the Ubiquitin-associated (UBA) Domain of Human Autophagy Receptor NBR1 and Its Interaction with Ubiquitin and Polyubiquitin. *J Biol Chem* **289**, 13890-13902 (2014).

5. Bedford, L. *et al*. Depletion of 26S proteasomes in mouse brain neurons causes neurodegeneration and Lewy-like inclusions resembling human pale bodies. *J Neurosci* **28**, 8189-8198, (2008).

6. Komatsu, M. *et al*. Loss of autophagy in the central nervous system causes neurodegeneration in mice. *Nature* **441**, 880-884, (2006).

7. Postic, C. *et al*. Dual roles for glucokinase in glucose homeostasis as determined by liver and pancreatic beta cell-specific gene knock-outs using Cre recombinase. *J Biol Chem* **274**, 305-315 (1999).

8. Tsuchiya, H., Tanaka, K. & Saeki, Y. The parallel reaction monitoring method contributes to a highly sensitive polyubiquitin chain quantification. *Biochem Biophys Res Commun* **436**, 223-229 (2013).
